# Decoding the association between platinum resistance and HPV status in cervical cancer using organoid models

**DOI:** 10.1186/s12915-026-02665-w

**Published:** 2026-06-26

**Authors:** Bin Zhang, Yao Lin, Suyao Xie, Xiaohong Zhu, Xinping Zhang, Lingyu Hu, Min Qiu, Hongkai Lee, Chongyang Shen, Mingliang You, Huayang Xing

**Affiliations:** 1 Department of Gynecology and Obstetrics, the First Affiliated Hospital, Fujian Medical University, Fuzhou, 350005 China; 2 Department of Gynecology, National Regional Medical Center, Binhai Campus of the First Affiliated Hospital, Fujian Medical University, Fuzhou, 350212 China; 3 Hangzhou AimingMed Technologies Co, Hangzhou, 310000 China; 4 The First Clinical College of Fujian Medical University, Fuzhou, 350005 China; 5 Basic Medicine School, Chengdu University of Traditional Chinese Medicine, Chengdu, 61000 China; 6 Hangzhou Cancer Institute, Hangzhou Cancer Hospital, Hangzhou, 310002 China; 7 Innovative Institute of Chinese Medicine and Pharmacy, Chengdu University of Traditional Chinese Medicine, Chengdu, 61000 China; 8 College of Biotechnology and Bioengineering, Zhejiang University of Technology, Hangzhou, 310032 China

**Keywords:** Organoid, Cervical cancer, HPV, Platinum resistance, Single-cell analysis, DNA repair

## Abstract

**Background:**

Cervical cancer remains a leading cause of cancer-related mortality in women, with high-risk HPV types (16, 18, 31, 33, 45, 52, and 58) playing a key role in its development. Platinum-based chemotherapy is standard for recurrent or metastatic cervical cancer, but platinum resistance remains a major challenge. The link between HPV infection and platinum resistance is not well understood. Tumor organoids offer a physiologically relevant model for investigating drug resistance mechanisms in cervical cancer.

**Results:**

We successfully established 37 cervical cancer organoids (CCOs), with 27 expanded for characterization and drug sensitivity testing. CCOs recapitulated the biomarker expression patterns and genomic features of their corresponding tissues. Drug sensitivity assays revealed increased cisplatin/carboplatin resistance in the non-HPV16 high-risk HPV CCOs subgroup, compared to HPV-negative CCOs. Single-cell RNA sequencing identified diverse cellular populations within CCOs, with platinum-resistant CCOs showing enhanced DNA repair, activation of the PI3K/AKT pathway, and suppressed apoptotic signaling. We also found that the PI3K/AKT inhibitor BYL719 enhanced the response to cisplatin and carboplatin in non-HPV16 high-risk HPV CCOs but not in HPV-negative CCOs.

**Conclusions:**

Cervical cancer organoids faithfully replicate the histopathological and genomic characteristics of their parental tumors, demonstrating their potential as preclinical models for studying cancer progression and drug resistance. Our findings suggest that the non-HPV16 high-risk HPV infections may contribute to increased platinum resistance through enhanced DNA repair mechanisms, increased PI3K/AKT activation and suppressed apoptosis, providing new insights for personalized cervical cancer treatment. The PI3K/AKT inhibitor BYL719 may serve as a potential therapeutic adjunct to enhance platinum efficacy in those patients with platinum-resistant cervical cancer.

**Supplementary Information:**

The online version contains supplementary material available at https://doi.org/10.1186/s12915-026-02665-w.

## Background

Cervical cancer is a significant cause of cancer-related mortality among women, despite advancements in screening, diagnosis, prevention, and treatment. The incidence of cervical cancer in China was approximately 11.4 per 100,000 women [1]. According to National Cancer Institute data, the prognosis for cervical cancer patients varies significantly depending on the stage at diagnosis. For those diagnosed at an early stage, the 5-year relative survival rate is 91%, compared to 19% for advanced or recurrent cervical cancer patients (https://www.cancer.gov/types/cervical/survival). Human papillomavirus (HPV) status plays a critical role in the development of cervical cancer [2]. The most well-known high-risk HPV types include HPV 16 and HPV 18, responsible for about 70% of all cervical cancer cases globally [2, 3]. Other notable high-risk types include HPV 31, 33, 45, 52, and 58 [2].

Chemotherapy is the standard treatment for recurrent or metastatic cervical cancer. The most commonly applied chemotherapeutic regimens for cervical cancer are the two platinum drug treatments (cisplatin and carboplatin) or a combination of the platinum drugs and paclitaxel, with a 29.1–67% overall response rate [4, 5]. However, platinum drug resistance in cervical cancer is a significant challenge in effective chemotherapy treatment [4]. Several mechanisms and strategies have been identified to understand and potentially overcome this resistance, including enhanced nucleotide excision repair (NER) pathway [6], platinum–DNA adducts formation [7], and increased expression of certain genes (such as *FGF13*) [8]. Combining platinum drugs with other agents [9] or molecular modifications of platinum compounds [10] have been applied to overcome the resistance while the outcome is not always promising.

Although various studies have explored the potential mechanisms of platinum drug resistance with cervical cancer cell lines [7, 8, 11] or mouse models [7, 12], the relationship between high-risk HPV infection and platinum resistance remains poorly understood. Recent development of organoid technology provides a promising approach to address this gap. Tumor organoids are three-dimensional cell culture models derived from patient tumor samples, replicating the original tumor's pathology and genomic mutations [13]. They have been widely used for personalized cancer research and drug testing [14], enabling more precise studies of disease mechanisms and therapeutic responses. Conventional cervical cancer cell lines like HeLa, CaSki or SiHa, have undergone extensive passaging, thus limiting their physiological relevance for preclinical testing. Compared to animal models, organoids are compatible with high-throughput drug screening, facilitating faster and more efficient drug discovery and testing [15]. In the past decade, female reproductive tract derived tissues or tumor organoids have been established, including uterine endometrium [16], trophoblast [17], ovarian [18], fallopian tubes [19, 20], and related cancers [21–24]. Therefore, tumor organoids offered a more physiological relevant platform for studying cancer biology and drug resistance in cervical and other gynecologic malignancies.

In this study, we established 37 cervical cancer organoids (CCOs) with surgical and biopsy samples from cervical patients and assessed cisplatin or carboplatin sensitivity across HPV-negative and HPV-positive groups. Additionally, we conducted single-cell analysis with established cervical organoids to explore potential platinum drug resistance mechanisms and their association with HPV status.

## Results

### Establishment of cervical cancer organoids biobank

**Fig. 1:**
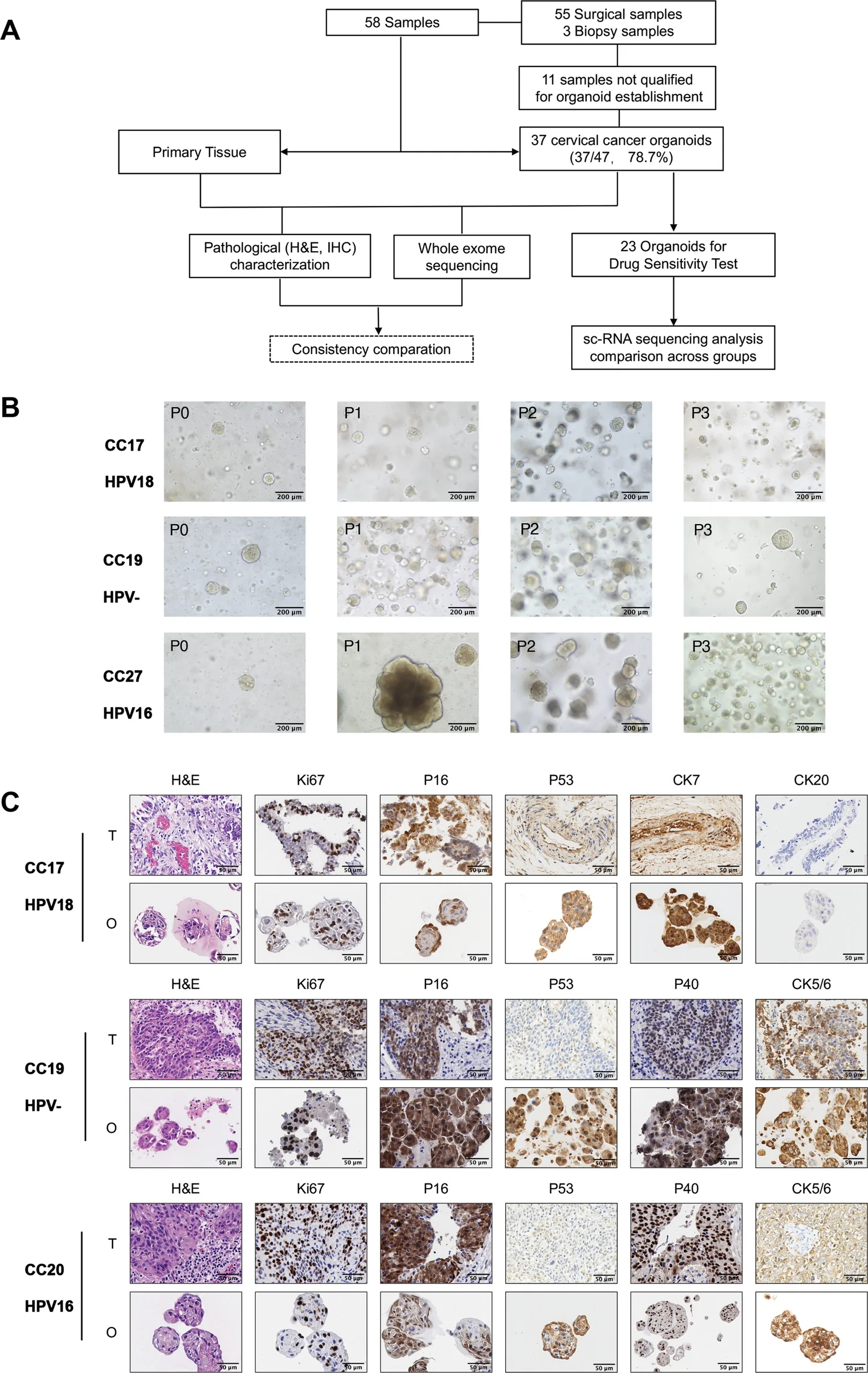
Establishment of cervical cancer organoids and histological characterization. **A** The flow diagram of this study. **B** Representative bright-field images showing the morphology of 1 HPV-negative CCO and 2 HPV positive CCOs. P stands for passage, Scale bar, 200 μm. **C** Histological comparison between CCOs and their parental tissues with H&E staining and immunohistochemistry staining of Ki67, P16, P40, P53, CK7, CK5/6, and CK20. Scale bar, 50 μm

In this study, 55 surgical and 3 biopsy samples were collected from 58 cervical cancer patients. Of these, 10 surgical samples and 1 biopsy sample (19.0%) were not processed for organoid establishment due to insufficient cell numbers (fewer than 3000 viable cells) following enzymatic dissociation. Out of the remaining 47 samples, 37 (78.7%) successfully gave rise to CCOs. The successful rate is comparable to the previous studies using CCOs [24, 25]. Among these, 27 CCOs (57.4%) were successfully expanded and stabilized for subsequent assays, i.e., histological characterization, drug response testing or genomic or single-cell transcriptomic analyses. Detailed information on these 27 CCOs is available in Additional file 4: Table S1. The full study design is shown in Fig. 1A.

The growth rates of cervical cancer organoids varied among different samples, and morphology differences in three-dimensional spheroid architecture were observed among organoids derived from different patients. Representative brightfield images of 1 adenocarcinoma (AC, CC17) sample and 2 squamous carcinoma samples (SCC, CC19, and CC27) are shown in Fig. 1B. The majority of the CCOs displayed a compact structure. To compare the histological characteristics with the original tissues, both organoids and their parental tissues were fixed, sectioned, and subjected to H&E staining, which revealed that the organoids closely resembled the histopathology of the corresponding tissues (Fig. 1C). Further analysis of marker expression revealed that the organoids and their primary tumors displayed comparable immunostaining patterns for Ki-67, p16, p40, p53, CK7, CK5/6, and CK20 (Fig. 1C). Specifically, the AC sample CC17 and its derived CCOs were Ki-67⁺, p16⁺, p53⁺, CK7⁺, and CK20⁻, whereas the SCC samples CC19 and CC20 and their corresponding CCOs were Ki-67⁺, p16⁺, p53⁺, p40⁺, and CK5/6⁺. Collectively, these findings indicate that CCOs are capable of recapitulating not only the morphological features but also the key biomarker expression patterns observed in cervical cancer.

### Genomic character concordance of cervical cancer organoids and corresponding tissues

**Fig. 2:**
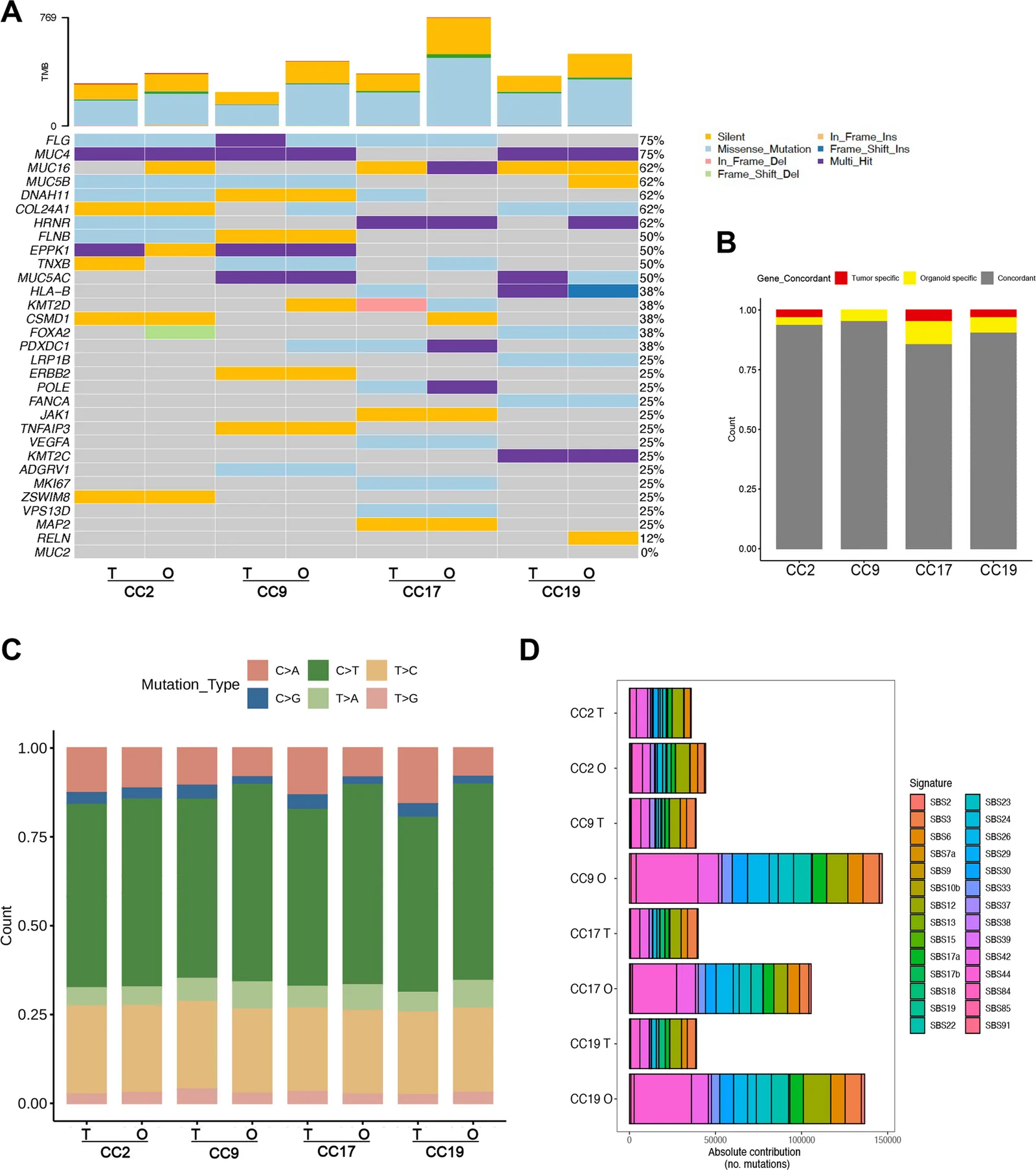
Genetic comparison between CCOs and their parental tissues. **A** Heatmap of gene mutation variations in the common mutated genes in cancers and the most frequently mutated genes of cervical cancer. Top, total mutation number of each sample. **B** Histogram showing the concordance (percent) of SNVs between CCOs and corresponding primary tumor tissues. **C** Bar graphs displaying the different contributions of point mutation types. **D** Total mutational load and mutational signatures of CCOs and paired tissues. Different colors represent 28 kinds of signatures

To validate the mutational concordance between CCOs and their corresponding tissues, we performed whole exome sequencing (WES) on three SCC samples (CC2, CC9, and CC19) and one AC sample (CC17). The gene list in Fig. 2A and Additional file 1: Figure S1 was curated based on high-frequency mutations reported in cervical cancer [26] and common mutations listed in OncoKB [27] and Cancer Genome Interpreter biomarker [28]. Mutations in the *FLG* gene, associated with skin disorders such as ichthyosis vulgaris, were identified in cervical cancer samples, a plausible finding [29]. Over 50% of all samples exhibited mutations in mucin family genes, including *MUC4*, *MUC5AC*, *MUC5B*, and *MUC16*, which are closely associated with glycosylation and metabolic diseases [30]. Additionally, mutations in cancer-related genes *KMT2D* and *KMT2C*, potential therapeutic targets for cervical cancer, were observed [31]. Beyond cervical cancer-specific mutations, alterations in genes such as *VEGFA*, *JAK1*, and *LRP1B*, commonly implicated in cancer [32–34] were detected in some samples. Of particular interest, mutations in HLA-B were present in 38% of samples (Fig. 2A), suggesting potential alterations in tumor antigen presentation that could influence the immune response against cancer [35].

Approximately 80–90% of gene mutation sites overlapped between the organoids and their corresponding tissues (Fig. 2B). The distribution of point mutation types was comparable between primary tissues and paired CCOs (Fig. 2C). The mutational spectrum exhibited a similar pattern across carcinoma samples (CC2, CC9, CC19) and adenocarcinoma (CC17), as well as across HPV statuses, including HPV16 (CC2, CC9), HPV-negative (CC19), and HPV18 (CC17), in both CCOs and parental tissues. Mutational signature analysis revealed that single-base substitutions (SBSs) in CCOs were consistent with those in the corresponding tissues (Fig. 2D). However, a higher number of mutations and SBSs were detected in organoids than corresponding tissues, potentially due to the enrichment of tumor cells in the organoids. Consistent with previous findings [36, 37], this suggests that the elevated mutations likely represent pre-existing, low-frequency variants in the original tumors.

### Chemosensitivities to cisplatin and carboplatin vary by HPV status

**Fig. 3:**
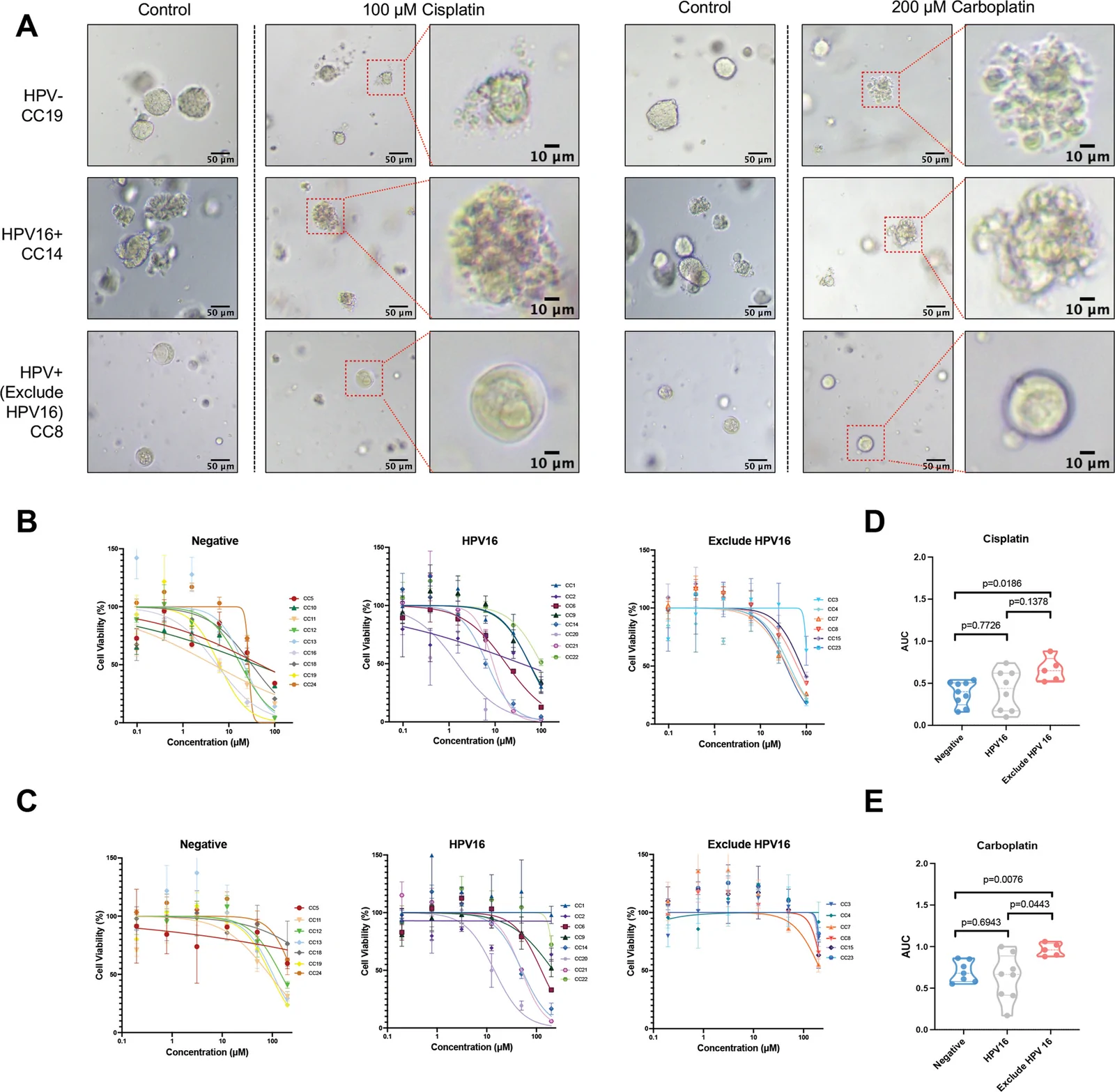
Responses of CCOs to cisplatin and carboplatin.** A** Brightfield images of CCOs treated with cisplatin and carboplatin respectively. Scale bar, 50 μm and 10 μm. The dose–response curves described the chemosensitivities of cisplatin (**B**) and carboplatin (**C**); 23 CCOs and 21 CCOs were included in the drug responses test for cisplatin and carboplatin, respectively. **D**, **E** AUC was calculated from the raw dose–response curves, normalized and displayed as a scatter dot plot. Pairwise comparison was performed using Mann–Whitney *U* test, and the *p*-values were FDR-adjusted

Cisplatin and carboplatin are commonly used chemotherapy drugs for cervical cancer, where platinum resistance frequently occurs. To investigate whether HPV infection affects platinum sensitivity, we performed cisplatin and carboplatin sensitivity assays on 23 CCOs. However, due to limited sample availability, two CCOs only had data for cisplatin sensitivity. Figure 3A and Additional file 2: Figure S2A illustrate that CCOs with different HPV subtypes display varying levels of cell viability following treatment with cisplatin or carboplatin. Notably, the representative images of HPV- CCOs and HPV16 CCOs exhibit a collapsing morphology after cisplatin or carboplatin administration, whereas the non-HPV16 high-risk HPV CCOs display markedly fewer morphological changes. The drug sensitivity curves for cisplatin (23 CCOs) and carboplatin (21 CCOs) are presented in Fig. 3B, C, respectively.

Consistent with prior study [38, 39], our findings confirmed that the relative area under the curve (AUC) is a more reliable metric for drug response than the half-maximal inhibitory concentration (IC50), particularly for resistant CCOs (Additional file 4: Table S1). A strong correlation between IC50 and relative AUC was observed for both cisplatin and carboplatin (Additional file 2: Figure S2B), further supporting the use of AUC as a robust measure of drug sensitivity in organoids. Notably, AUC values of both cisplatin and carboplatin were significantly higher in HPV-positive groups when the HPV16 subgroup was excluded (labeled as Exclude HPV16) (Table 1, Fig. 3D, E). One sample was excluded in the comparison due to its co-infection with HPV16 and HPV52 (Table 1, Fig. 3D, E). Interestingly, no significant differences were observed between the HPV-negative and HPV16 groups, and across the different histology groups (Table 1).

**Table 1:** Statistic comparison of normalized AUC values across HPV status and histology groups. Data was presented in median and min-max of the values. The target HPV status versus the rest of groups was compared using Mann-Whitney *U* test, with *p*-values adjusted with FDR

	**Normalized AUC Median, Min-Max (N, FDR-q)**	
**Parameters**	**Cisplatin**	**Carboplatin**
*HPV status*		
Negative	0.40, 0.16–0.54 (9, 0.1631)	0.68, 0,55-0.86 (7, 0.3114)
HPV16	0.44, 0.10–0.74 (8, 0.5847)	0.67, 0.17–1.00 (8, 0.2353)
Exclude HPV16	0.65, 0.52–0.88 (5, 0.0325)	0.96, 0.88–1.06 (5, 0.0074)
*Histology*		
Squamous carcinoma	0.52, 0.10–0.88 (13, 0.7241)	0.74, 0.17–1.06 (12, 1.0000)
Adenocarcinoma	0.52, 0.19–0.71 (3, 0.8230)	0.96, 0.90–1.02 (2, 0.2956)
Others*	0.35, 0.30–0.40 (3, 0.7206)	0.61, 0.55–0.68 (3, 0.2956)

*Included 1 Endometrioid carcinoma, 1 Adenosarcoma, and 1 Endometrioid carcinoma + Squamous carcinoma samples

### Single-cell transcriptome landscape of cervical cancer organoids

**Fig. 4:**
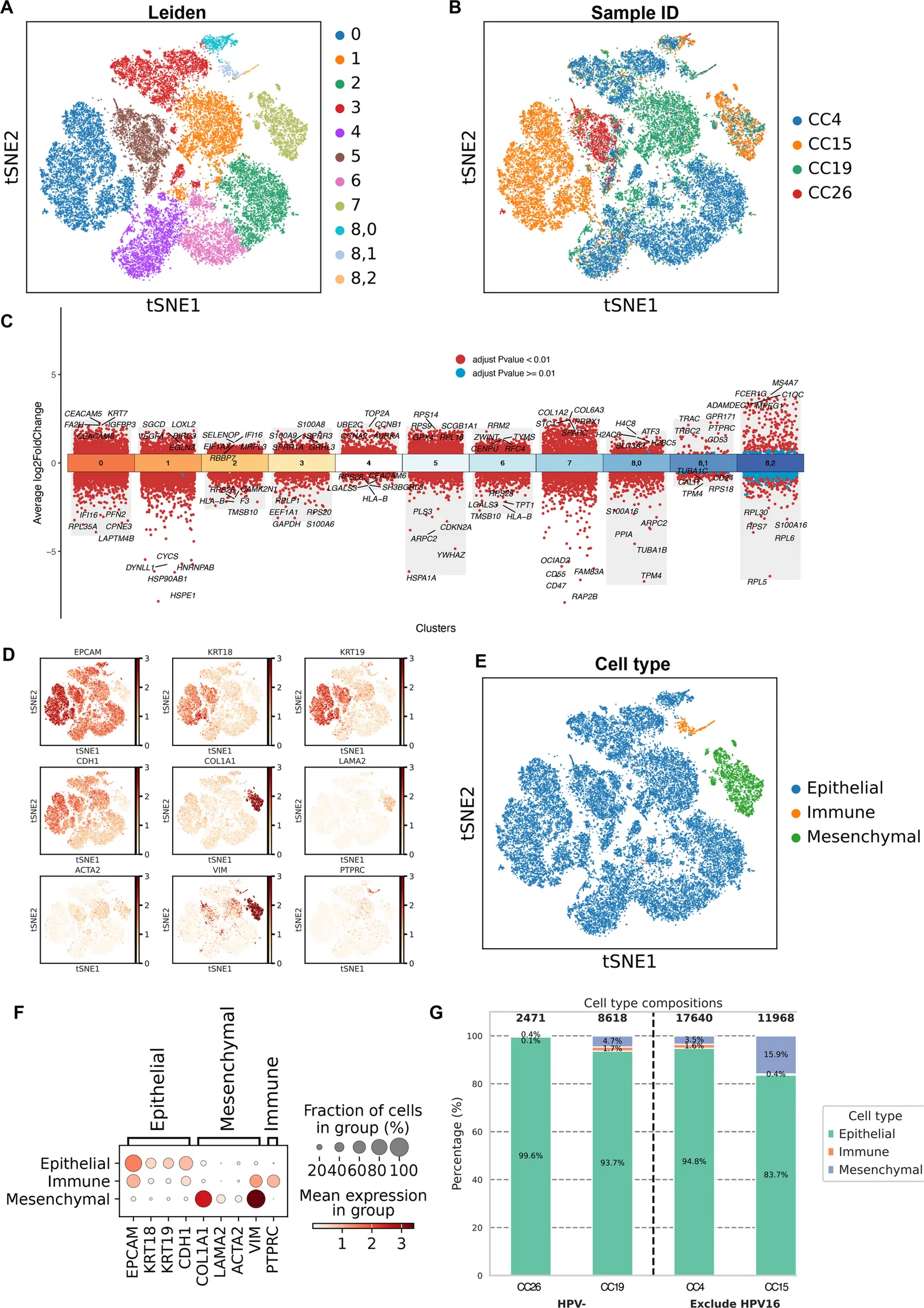
Construction of single-cell expression atlas for CCOs varies by HPV status.** A** t-SNE plot showing four distinct cellular clusters identified from 40,697 cells, with each dot representing a single cell. **B** t-SNE plots displaying the clustering of cells by individual sample. **C** Top five upregulated and downregulated genes identified in each cellular cluster. **D** t-SNE plots showing the expression and spatial distribution of markers for epithelial cells (*EPCAM, KRT18, KRT19, CDH1*), mesenchymal cells (*COL1A1, LAMA2, ACTA2, VIM*), and immune cells (*PTPRC*). **E** t-SNE plot categorizing the cells into three major types. **F** Bubble diagram illustrating the canonical marker genes across different cell subtypes. The *X*-axis represents marker genes, and the *Y*-axis represents distinct cell subtypes. **G** Bar chart showing the proportions of each cell type across the four samples

As a significant difference in platinum sensitivity was observed between the HPV-negative and non-HPV16 high-risk HPV CCOs groups, scRNA-seq was performed on four CCOs from the 2 groups to investigate cellular composition and pathway alterations associated with platinum resistance. The analysis included two HPV-negative CCOs (CC19 and CC26) and two HPV-positive CCOs (HPV33 and HPV39 for CC4 and CC15, respectively). To minimize potential passage-related effects, all organoids used for the scRNA-seq analysis were within passage 5. After quality control and filtering, a total of 40,697 cells were retained for downstream analysis. These cells were grouped into ten distinct clusters and visualized using t-SNE (Fig. 4A). The cell numbers for each sample were as follows: 17,640 (CC4), 11,968 (CC15), 8618 (CC19), and 2471 (CC26).

Distinct clustering patterns were observed for each sample, highlighting inter-sample heterogeneity (Fig. 4B). The top five upregulated and five downregulated genes in each cluster were identified and are shown in Fig. 4C. These cells were then annotated to three major cell types following the canonical marker genes: epithelial cell (*EPCAM, KRT18, KRT19, CDH*1), mesenchymal cell (*COL1A1, LAMA2, ACTA2, VIM*), and immune cell (*PTPRC*) (Fig. 4D–F, Additional file 5: Table S2). The proportions of each cell type across the four samples are summarized in Fig. 4G.

Overall, epithelial cells constituted the majority cell type in all CCOs, accompanied by smaller fractions of mesenchymal cells and a minor population of immune cells (Fig. 4E, G). Interestingly, a notable increase in mesenchymal cells was observed in one HPV-positive CCO (CC15). This finding suggested that the mesenchymal population may be enriched in the platinum-resistant group, potentially reflecting a role for mesenchymal cells in platinum resistance. However, sample heterogeneity should also be considered as a possible contributing factor to this observation. In conclusion, these results demonstrated that CCOs exhibit diverse cellular compositions, including epithelial and mesenchymal cell populations.

### Single-cell sequencing reveals mechanisms of platinum resistance associated with HPV status

Cisplatin and carboplatin inhibit cervical cancer progression by inducing DNA cross-linking, thereby disrupting DNA replication and triggering apoptosis [4]. Additionally, they generate reactive oxygen species (ROS), which enhance cytotoxic effects [40]. However, their therapeutic efficacy is often compromised by resistance mechanisms such as enhanced DNA repair, increased drug efflux, and alterations in apoptosis pathways [4, 41].

**Fig. 5:**
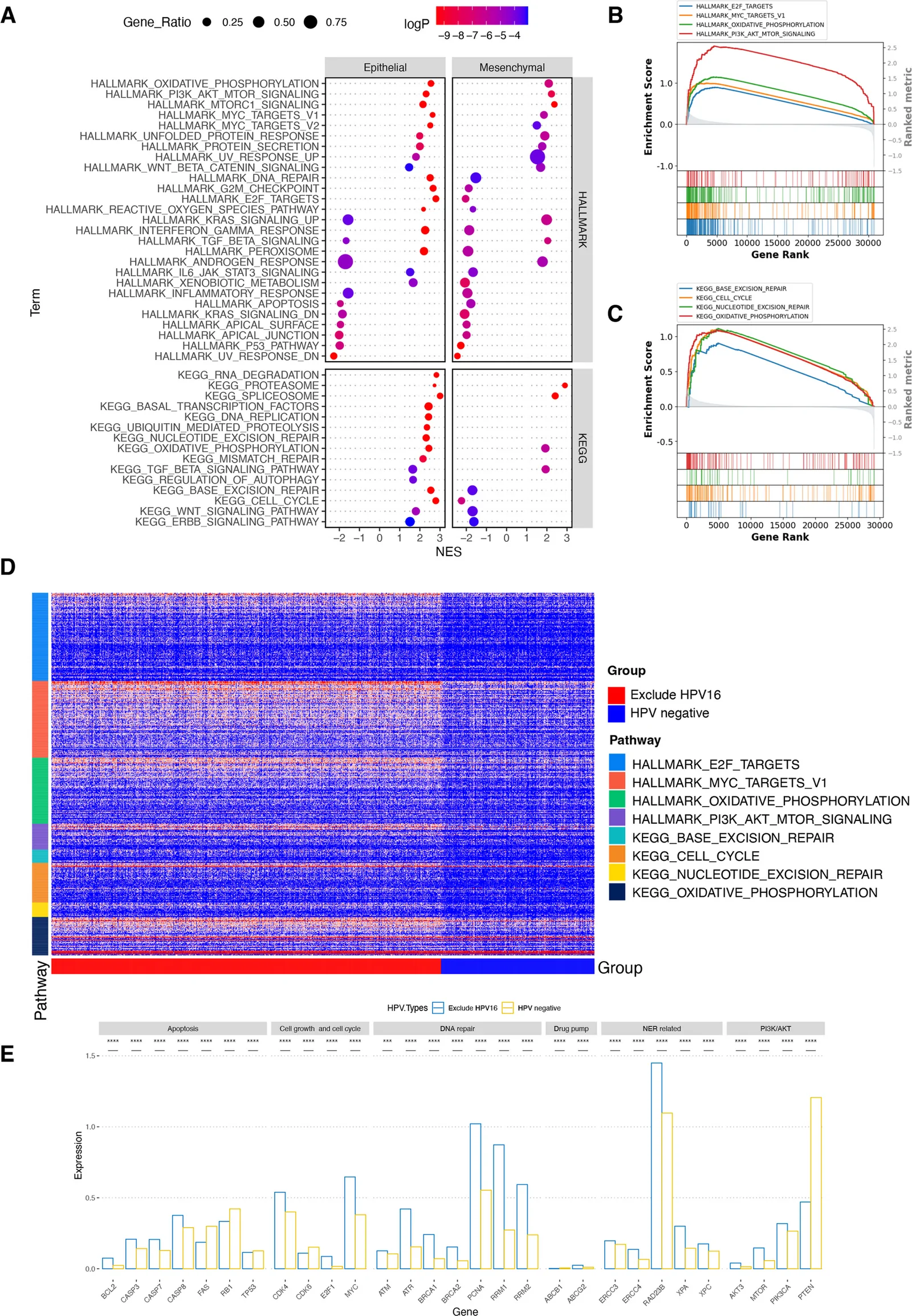
Transcriptome differences between HPV-negative and Exclude HPV16 group. Representative enrichment plot of GSEA (**A**) hallmark and KEGG pathways of the Exclude HPV16 group compared to the HPV-negative group, obtained from epithelial and mesenchymal cells, respectively. GSEA plots of selected **B** hallmark pathways, **C** KEGG pathways, and **D** heatmaps of lead gene expression in Myc targets V1, oxidative phosphorylation, PI3K-AKT-mTOR, E2F targets, base excision repair, cell cycle, nucleotide excision repair of the Exclude HPV16 group compared to HPV-negative group, obtained from epithelial cells. **E** Bar graph of selected genes expression level of Exclude HPV16 group compared to the HPV-negative group, obtained from epithelial cells. Pairwise comparison was performed using Mann–Whitney *U* test, ***p < = 0.001, ****p < = 0.0001

To further elucidate the mechanisms underlying platinum-based chemotherapy resistance in the non-HPV16 high-risk HPV group, we performed gene set enrichment analysis (GSEA) on hallmark and Kyoto Encyclopedia of Genes and Genomes (KEGG) gene sets/pathways available in Molecular Signatures Database [42] in epithelial and mesenchymal cells. As shown in Fig. 5A, both epithelial and mesenchymal cells exhibited decreased p53 and apoptosis pathways, enrichment in oxidative phosphorylation, PI3K-AKT-mTOR signaling, mTORC1 signaling, Myc targets V1/V2, the unfolded protein response, and Wnt/β‐catenin signaling (Fig. 5A, B, and Additional file 3: Figure S3A). Additionally, specific pathways, including DNA repair, the G2M checkpoint, E2F targets, the ROS pathway, peroxisome function, interferon gamma response, IL6-JAK-STAT3 signaling, and xenobiotic metabolism, were upregulated in epithelial cells but not in mesenchymal cells in the non-HPV16 high-risk HPV group (Fig. 5A). Further, KEGG analysis showed elevated NER, base excision repair (BER), and cell cycle in epithelial cells. In general, more dysregulated pathways were found in epithelial cells than mesenchymal cells (Fig. 5A).

Several of these pathways are closely associated with key cellular processes. For example, p53, E2F targets, Myc targets V1/V2, and the G2M checkpoint are integral to cell cycle regulation, apoptosis and proliferation [43–46]. In contrast, oxidative phosphorylation, the ROS pathway, mTORC1 signaling, peroxisome function, and the unfolded protein response primarily mediate cellular stress responses [47–49]. Notably, PI3K-AKT-mTOR and Wnt/β-catenin signaling are well-established contributors to drug resistance in cancer progression [50, 51]. DNA repair, NER, BER are generally associated with DNA repair capacity, which allows cancer cells to more effectively repair DNA damage induced by platinum drugs [52, 53].

Given that the predominant cell type in CCOs is epithelial, we further elaborated the enrichment of Myc targets V1, oxidative phosphorylation, PI3K-AKT-mTOR, E2F targets, BER, NER, and cell cycle in epithelial cells through enrichment score analysis and lead gene expression (Fig. 5B–D). Moreover, a few key genes’ expressions were compared in the related pathways, including apoptosis, cell growth and cell cycle, DNA repair, NER, drug pump efflux, and PI3K/AKT pathway (Fig. 5E). Collectively, the resistant CCOs group exhibited the increased DNA repair processes, decreased apoptosis, acceleration of cell cycle progression, and activation of the PI3K-AKT-mTOR in the epithelial cells.

Enrichment score analysis plots of mesenchymal cells were listed in Additional file 3: Figure S3. Interestingly, basal cell carcinoma pathway and endometrial cancer pathway were significantly elevated in mesenchymal cells but not found in epithelial cells. These pathway activations may reflect HPV-induced epithelial–mesenchymal crosstalk and stromal activation. Given the increased mesenchymal cell population were observed in one HPV-positive organoid (CC15, Fig. 4G), this conclusion may need further validation with a larger cohort.

### PI3K/AKT inhibition by BYL719 enhances CCOs’ sensitivity to platinum drugs

**Fig. 6:**
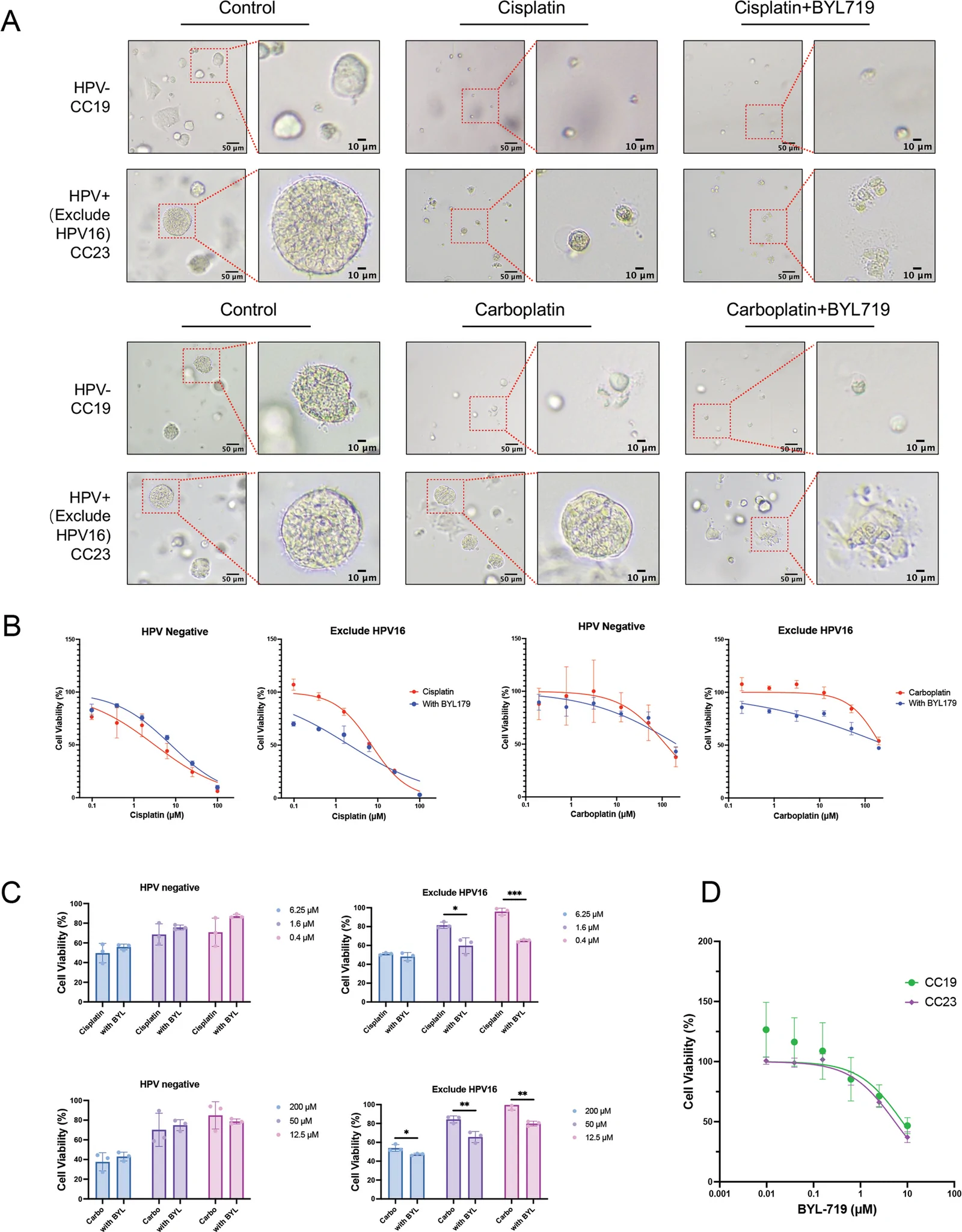
BYL719 enhances CCOs’ sensitivity to cisplatin/carboplatin in Exclude HPV16 group. **A** Brightfield images of CCOs treated with cisplatin or carboplatin, with or without 1 μM BYL719. Scale bars: 50 μm and 10 μm. **B** Dose–response curves of CC19 and CC23 treated with cisplatin or carboplatin in the presence (blue) or absence (red) of 1 μM BYL719. **C** Comparison of cell viability in CC19 and CC23 following cisplatin (upper panel) or carboplatin (lower panel) treatment, with and without 1 μM BYL719, at selected concentrations. **D** Dose–response curves of CC19 and CC23 treated with BYL719 alone. Pairwise comparison was performed with unpaired *t*-test. *p < = 0.05, **p < = 0.01, ***p < = 0.001

Given that activation of the PI3K/AKT pathway was observed in both epithelial and mesenchymal cells of platinum-resistant CCOs, we next examined whether pharmacological inhibition of this pathway could enhance their sensitivity to cisplatin and carboplatin. BYL719 (Alpelisib), an FDA-approved, α-specific PI3K inhibitor for PIK3CA-mutated advanced or metastatic breast cancer, was used to inhibit PI3K/AKT signaling in this study. As expected, BYL719 significantly enhanced the efficacy of both cisplatin and carboplatin in the non-HPV16 high-risk HPV CCOs (Fig. 6A), with the effect being most pronounced at lower platinum concentrations (Fig. 6B, C), indicating a BYL719-induced hypersensitization to platinum agents. In contrast, this hypersensitization effect was not observed in HPV-negative CCOs (Fig. 6B, C), and BYL719 alone showed comparable effects across both groups (Fig. 6D). These findings indicate that inhibition of the PI3K/AKT pathway can attenuate platinum resistance and suggest the potential clinical utility of BYL719 in platinum-resistant cervical cancer patients.

## Discussion

Resistance to cisplatin and carboplatin is a significant challenge in the chemotherapy of cervical cancer [4]. In parallel, HPV infection is strongly linked to the development of cervical cancer, which has been observed in a patient-derived organoid model [54]. However, the relationship between HPV infection and platinum resistance in cervical cancer remains poorly understood. To address this gap, we established an organoid biobank from 58 cervical cancer patient samples. Of these, 47 underwent processing, and 37 gave rise to CCOs, with 27 CCOs successfully expanded and stabilized for downstream analyses. The successful rate of generating organoids is comparable to the prior study [24, 25]. Next, the consistency of CCOs with their corresponding parental tissues was validated through H&E, IHC, and WES. These analyses confirmed that CCOs replicate the molecular and genomic profiles of the parental tumors, making them ideal candidates for disease modeling and drug testing.

Mutational profiling of CCOs revealed a high degree of concordance with primary tumors, with over 80% of mutation sites overlapping. This suggested that CCOs faithfully preserve the genetic landscape of the parental tumors, making them useful for mutational analyses. Key mutations in cervical cancer-related genes, such as *KMT2D*, *KMT2C*, and the mucin family genes, were detected, confirming the genetic fidelity of CCOs. Additionally, alterations in HLA-B and other immune-related genes suggested that immune evasion mechanisms may influence responses to immune-based therapies [35]. Notably, previous studies have demonstrated that patient-derived organoids (PDOs) in other cancers, such as pancreatic and lung cancers [36, 37], exhibit uncovered key driver mutations and a greater mutational burden, aiding in the identification of clinically relevant genomic alterations. Our findings similarly showed a higher number of mutations and SBSs in CCOs, which may be attributed to the higher purity of tumor cells in the organoids. These results underscored the utility of organoid models in exploring genetic and immune landscape alterations in cervical cancer.

Previous studies have shown elevated resistance to Adriamycin-Cyclophosphamide and paclitaxel regimens in HPV-positive breast cancer cohorts, possibly through disruption of genes involved in cell death [55]. However, the mechanisms by which HPV infection influences platinum resistance are still unclear. Interestingly, we observed increased resistance to cisplatin and carboplatin in non-HPV16 high-risk HPV CCOs, but not in HPV16 CCOs. These findings suggested that high-risk HPV infection, but not HPV16, may be linked to platinum resistance in cervical cancer. One possible explanation for the differential platinum sensitivity between HPV16 and other high-risk HPV types may lie in the higher intrinsic immunogenicity of HPV16. One study suggested that HPV16 elicits stronger antiviral immune responses than HPV18 [56], which could contribute to greater chemosensitivity through enhanced immune-mediated cytotoxicity. In addition, although all high-risk HPV types increase E6 and E7 oncoproteins, the efficiency and regulatory balance of these interactions vary by genotype. Evidence regarding these differences remains mixed: one study reported that E6 and E7 from HPV18 are less efficient at inhibiting Toll-like receptor 9 (TLR9) transcription than those from HPV16 [57]; whereas another found higher viral integration frequencies in HPV18 compared to HPV16 [3], potentially leading to stronger activation of DNA repair and pro-survival pathways in non-HPV16 high-risk HPV cervical cancer patients. However, considering the sample number of this cohort, it will be worthwhile to validate this finding with a larger sample size.

Cisplatin and carboplatin exert their cytotoxic effects by forming platinum–DNA adducts, which bind to DNA and induce crosslinking, distorting the DNA structure [4]. This leads to inhibition of transcription and DNA replication, ultimately triggering apoptosis through pathways such as p53 [4, 58]. Previous studies found that HPV E6 and E7 proteins degrade the tumor suppressors p53 and pRb, respectively [59], which are critical for apoptosis and cell cycle regulation. GSEA revealed downregulation of the p53 and apoptosis pathway in non-HPV16 high-risk HPV CCOs in both epithelial and mesenchymal cells. In terms of apoptosis-related markers, *BCL2* was significantly upregulated, while *FAS*, *RB1*, and *TP53* were significantly downregulated in HPV-positive (exclude HPV16) CCOs. Interestingly, *CASP3*, *CASP7*, and *CASP8* were elevated in epithelial cells, suggesting potential compensatory mechanisms. RB1 deficiency is known to weaken p53 activation and upregulate DNA repair pathways, both of which contribute to platinum resistance [60].

Additionally, dysregulation of cell cycle progression, proliferation, DNA repair, and stress response pathways was observed, aligning with previous findings that platinum resistance in cervical cancer arises from enhanced DNA repair, altered apoptotic pathways, and increased cellular stress [4]. Notably, NER, BER, and mismatch repair (MMR), particularly NER, play crucial roles in removing platinum-induced DNA lesions, thereby increasing resistance [52]. Key DNA repair genes such as *ATM*, *ATR*, *BRCA1/2*, *PCNA*, *RRM1/2*, *ERCC3/4*, *XPA*, and *XPC* were significantly upregulated in non-HPV16 high-risk HPV CCOs compared to HPV-negative CCOs, suggesting that elevated DNA repair activity is a major contributor to platinum resistance in this subgroup. In addition, the HPV E7 oncoprotein binds to and inactivates the pRb protein, leading to the release and activation of the E2F transcription factor, a key regulator of cell cycle progression and DNA replication [61]. Notably, E2F activation has been reported to enhance cisplatin resistance in ovarian cancer [62], consistent with our observation of elevated E2F signaling in the platinum-resistant CCOs. However, inclusion of the HPV16 group in future comparative analyses may yield deeper insights into the molecular mechanisms contributing to elevated platinum resistance.

A previous study showed that HPV-infected cells exhibit overexpression of *APOA1*, which may contribute to resistance through activation of survival pathways such as p38 *MAPK* and *PI3K* [63]. Consistently, we observed upregulation of the PI3K/AKT pathway in both epithelial and mesenchymal cells, which is increasingly recognized for its role in cisplatin resistance [64, 65]. Furthermore, inhibition of the PI3K/AKT pathway with BYL719 enhanced the sensitivity of non-HPV16 high-risk HPV CCOs to cisplatin and carboplatin, particularly at lower concentrations. BYL719, also known as Alpelisib, is an FDA-approved PI3Kα inhibitor for the treatment of breast cancer. It has also demonstrated significant efficacy in *PIK3CA*-mutated, recurrent or advanced cervical cancer [66]. Previous studies have reported that BYL719 can overcome paclitaxel resistance in cervical cancer [67] and synergize with cisplatin in *PIK3CA*-mutated SKOV-3 [64] and nasopharyngeal carcinoma cell lines [65]. Interestingly, in our study, this hypersensitization effect was observed only in non-HPV16 high-risk HPV CCOs, but not in HPV-negative CCOs, providing evidence that targeting the PI3K/AKT pathway may offer therapeutic potential for enhancing platinum sensitivity and improving clinical outcomes in non-HPV16 high-risk HPV-associated cervical cancer. Nevertheless, these findings should be validated in a larger cohort, and the underlying molecular mechanisms warrant further investigation.

## Conclusion

In summary, our study demonstrated that cervical cancer organoids are a reliable and reproducible model for investigating cervical cancer biology and drug responses. The establishment of these organoid models from diverse HPV statuses and histological subtypes provides a unique opportunity to explore the impact of HPV infection on drug resistance and the underlying molecular mechanisms. Identifying key signaling pathways involved in platinum resistance offers valuable insights that could guide the development of novel therapeutic strategies to overcome chemoresistance in cervical cancer. Furthermore, we highlight the PI3K/AKT inhibitor BYL719 as a potential therapeutic adjunct capable of enhancing platinum efficacy in the non-HPV16 high-risk HPV patients with platinum-resistant cervical cancer.

## Materials and methods

### Collection of human specimens and HPV testing

The collection of cervical cancer patient samples and the associated experiments were reviewed and granted approval by the Ethics Committee of the First Affiliated Hospital of Fujian Medical University (MRCTA, ECFAH of FMU [2021]435). Primary cervical cancer biopsies and clinical data were procured with the informed consent of each participant within the department.

HPV testing of the clinical samples was performed with HPV nucleic acid genotyping detection kit (Tellgen, Shanghai, China). Genotyping was performed using a Luminex 200 flow fluorescence analyzer, following manufacturer’s instructions. This assay covered 27 HPV genotypes, including 17 high-risk subtypes (16, 18, 26, 31, 33, 35, 39, 45, 51, 52, 53, 56, 58, 59, 66, 68, and 82) and 10 low-risk subtypes (6, 11, 40, 42, 43, 44, 55, 61, 81, and 83).

### Cervical cancer patient-derived organoids (CCOs) culture

The protocol for establishing CCOs was refined from a previously published method [68]. Cervical cancer tissues were preserved in pre-cold MasterAim® Tissue Preservation Medium (AimingMed, China) and transported to the lab within 48 h. Tissues were washed in cold DPBS with 1% penicillin/streptomycin, minced, and enzymatically digested in MasterAim® Tissue Digestion Buffer I (AimingMed) at 37 °C for 30 min. The digested fragments were filtered through a 100 μm strainer to obtain a single-cell suspension, centrifuged, and resuspended in Matrigel. The cell-Matrigel mixture (50 µL) was seeded into 24-well plates, overlaid with 500 µL of MasterAim® Cervical Cancer Organoid Medium (AimingMed), and maintained with medium changes every 3–4 days. Organoids were passaged with TrypLE Express (Thermo, US) biweekly at a 1:3 to 1:5 dilution based on size and density. All reagents and consumables used in this study are listed in Table S3.

### H&E and IHC staining of tissues and organoids

Cervical cancer tissues and organoids (at least 3 × 10^5^ cells were used per sample) were fixed in 4% paraformaldehyde at 4 °C for 24 h, then dehydrated and embedded in paraffin for sectioning. Fixed organoids were mixed with 30 μL of preheated 3% agarose, allowed to solidify, and transferred to embedding cassettes. Samples were dehydrated through graded ethanol and xylene, followed by paraffin infiltration and embedding. Paraffin blocks were cooled, removed, and trimmed for sectioning. Sections were stained with hematoxylin and eosin (H&E) for morphology or underwent immunohistochemistry (IHC) to detect Ki-67, P16, P40, p53, CK7, CK5/6, and CK20. For IHC, antigen retrieval, blocking, and incubation with primary antibodies (Table S3) were performed overnight at 4 °C, followed by secondary antibody incubation for 30 min at room temperature, counterstaining, dehydration, and mounting for analysis.

### Platinum-based chemotherapy drug sensitivity test for CCOs

CCOs were digested with TrypLE Express (Thermo, USA), resuspended in 60% Matrigel at 100 cells/μL, and 10 μL of the suspension (1000 cells/well) was plated in 384-well plates. After the Matrigel solidified, 45 μL of culture medium was added, and cells were cultured for 48 h before drug testing. Cisplatin and carboplatin were tested at six concentrations (cisplatin, 100–0.098 μM; carboplatin, 200–0.195 μM, fourfold dilution) for 72 h. The combination assays were conducted by co-treating CCOs with 1 μM BYL719 and varying concentrations of cisplatin or carboplatin. After treatment, 45 μL of CellTiter-Glo Luminescent Cell Viability Reagent (Promega, USA) was added per well, and luminescence was measured using a Tecan Infinite M200 PRO plate reader.

### Whole exome sequencing and data analysis

DNA was captured using Agilent SureSelect Human All Exon V6 chip and sequenced on Illumina Novaseq 6000 (Illumina, USA). The sequence reads were mapped to the genome (hg38) using BWA-MEM [69]. Subsequently, GATK [70] was employed for somatic mutation detection without control data, with potential germline mutations filtered using two approaches: the panel-of-normals and the germline resource, to generate the final mutation detection results. The identified mutations were then annotated using ANNOVAR [71], followed by aggregation, statistical analysis, and oncoplot generation using MAFtools [72], while MutationalPatterns [73] was applied for mutation frequency and feature analysis.

### Single-cell RNA sequencing and data processing

Library preparation was performed using the BD Rhapsody system and Whole Transcriptome Analysis (WTA) Amplification Kit (BD Biosciences, NJ, USA; 633,801) following the manufacturer’s guidelines. Library concentration and fragment distribution were assessed with the Qubit™ dsDNA HS Assay Kit (Thermo Fisher, USA) and D1000 ScreenTape, respectively, while molar concentration was determined using the KAPA Library Quant Kit (Illumina). Sequencing was carried out on the NovaSeq XPlus platform (Illumina) with the NovaSeq S4 Reagent Kit.

Sequencing data were pre-processed using the BD Rhapsody Pipeline [74] (version 2.0) with default parameters to generate a single-cell expression matrix, utilizing pre-built human Gencode V43 WTA references from the BD Biosciences website. Downstream analyses, performed using Scanpy (version 1.9.6) [75], included initial quality control to retain cells expressing at least 200 genes and genes detected in at least three cells, while excluding cells with more than 4500 expressed genes or over 20% mitochondrial gene content or over 25,000 total expression count. Gene expression data were normalized using default parameters, and the 2000 most variable genes were identified using Scanpy’s “highly_variable_genes” method, applying the “seurat_v3” flavor. Principal component analysis (PCA) was performed, followed by batch effect correction using Harmony integration (implemented via Scanpy [75]) on a per-sample basis. The top 15 PCs corrected with Harmony were used for t-distributed Stochastic Neighbor Embedding (t-SNE) and Leiden clustering (15 neighbors and resolution setting of 0.2). Finally, gene set enrichment analysis was conducted using GSEApy [76].

### Statistical analysis

For comparative analyses, statistical significance was assessed using Mann–Whitney *U* test with *p*-values adjusted with False Discovery Rate–Benjamini–Hochberg method using Scipy and Statsmodels packages available in Python. A threshold of adjusted *p* < 0.05 was set to denote statistical significance.

## Supplementary Information

Additional file 1: Figure S1 (related to Figure 2). Genomic concordance of CCOs compared with corresponding tissues. Supplemented mutation landscape of SNVs in CCOs and paired tissues.

Additional file 2: Figure S2 (related to Figure 3). Supplementary data of CCOs’ responses to cisplatin and carboplatin. (A) Brightfield images of CCOs treated with cisplatin and carboplatin respectively. Scale bar, 50 μm and 10 μm. (B) Correlation between relative AUC and IC50 values. IC50 values greater than twofold the highest tested concentration were excluded. Correlation coefficient and p value was determined by Pearson correlation.

Additional file 3: Figure S3 (related to Figure 5). Transcriptome differences analysis in mesenchymal cells. Enrichment score analysis plots of selected hallmark pathways and KEGG pathways of Exclude HPV16 group compared to HPV negative group, obtained from mesenchymal cells.

Additional file 4: Table S1. Clinical information and drug sensitivity characteristics of the cervical cancer organoids used in this study. IC50 values of carboplatin greater than twofold the highest tested concentration (200 μM) were showed as >400 μM.

Additional file 5: Table S2 (related to Figure 4). Differential expression genes and the assigned cell types of the 9 cell clusters identified from the 40,697 cells sequenced in this study.

Additional file 6: Table S3. Reagents and consumables used in this study

## Data Availability

The sequencing data generated in this study have been deposited in the The raw data was submitted to the National Genomics Data Center (NGDC) Genome Sequence Archive (GSA) database under accession number HRA012221 [79]. The data are available according to GSA policies.

## Abbreviations

AC: Adenocarcinoma
AUC: Area under the curve
BER: Base excision repair
CCO: Cervical cancer organoid
EMT: Epithelial-to-mesenchymal transition
GSEA: Gene set enrichment analysis
HPV: Human papillomavirus
H&E: Hematoxylin and eosin
IC50: Half-maximal inhibitory concentration
IHC: Immunohistochemistry
KEGG: Kyoto Encyclopedia of Genes and Genomes
MMR: Mismatch repair
NER: Nucleotide excision repair
PCA: Principal component analysis
PDO: Patient-derived organoid
ROS: Reactive oxygen species
scRNA-seq: Single-cell RNA sequencing
SCC: Squamous carcinoma
SNVs: Single-nucleotide variants
t-SNE: T-distributed Stochastic Neighbor Embedding
TLR9: Toll-like receptor 9
TMB: Tumor mutational burden
WES: Whole exosome sequencing
WTA: Whole Transcriptome Analysis
