# Supplementary material for: Decoding the association between platinum resistance and HPV status in cervical cancer using organoid models
Source: BMC Biol. 2026 Jun 26;24:197. doi: 10.1186/s12915-026-02665-w (PMC13591797; doi:10.1186/s12915-026-02665-w)
Supplement: Supplementary file 1 [file 12915_2026_2665_MOESM1_ESM.pdf]

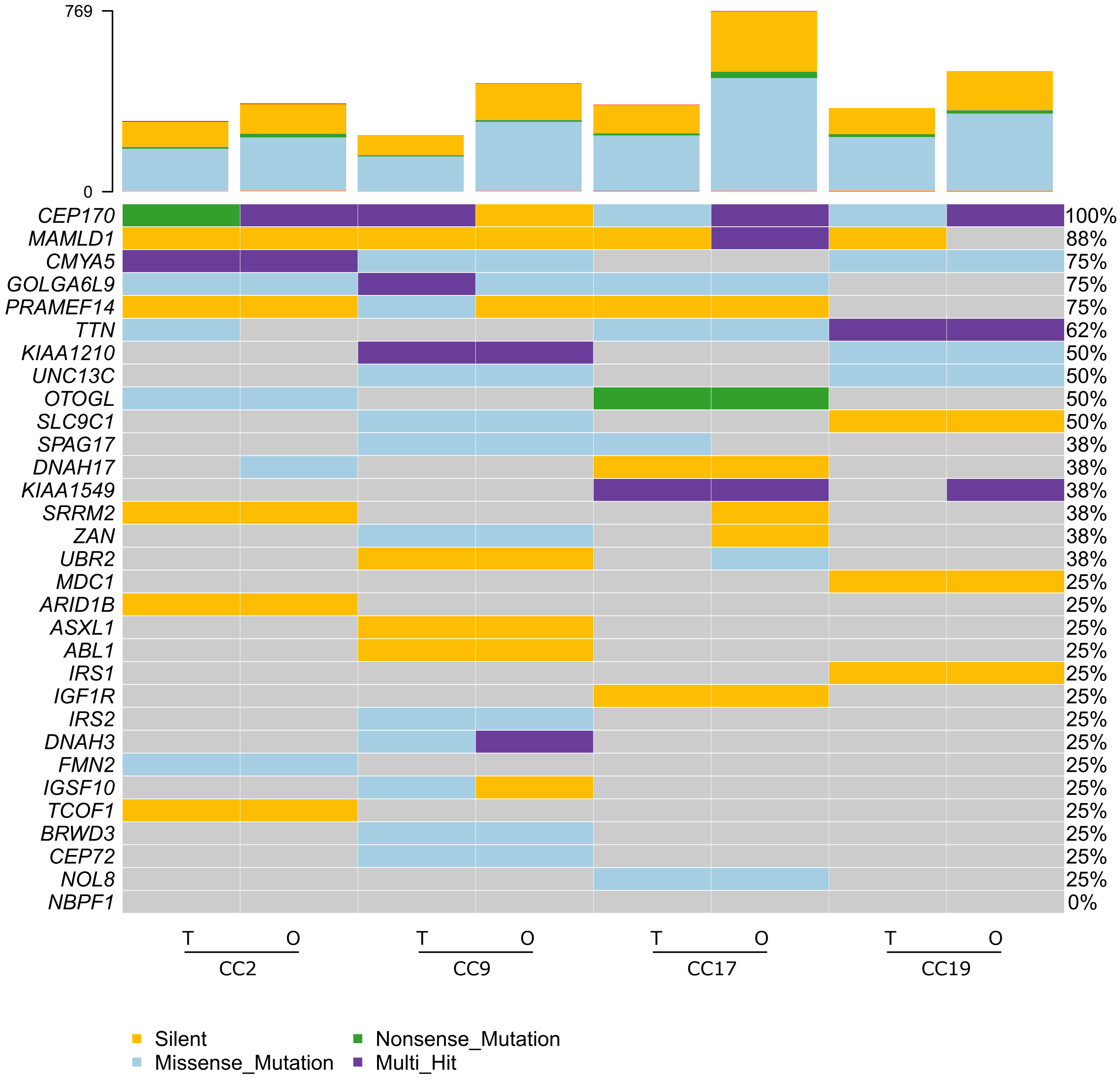
769
0
CEP170
MAMLD1
CMYA5
GOLGA6L9
PRAMEF14
TTN
KIAA1210
UNC13C
OTOGL
SLC9C1
SPAG17
DNAH17
KIAA1549
SRRM2
ZAN
UBR2
MDC1
ARID1B
ASXL1
ABL1
IRS1
IGF1R
IRS2
DNAH3
FMN2
IGSF10
TCOF1
BRWD3
CEP72
NOL8
NBPF1
100%
88%
75%
75%
75%
62%
50%
50%
50%
50%
38%
38%
38%
38%
38%
38%
25%
25%
25%
25%
25%
25%
25%
25%
25%
25%
25%
25%
25%
25%
0%
T O
CC2
T O
CC9
T O
CC17
T O
CC19
Silent
Missense_Mutation
Nonsense_Mutation
Multi_Hit
