# Supplementary material for: Decoding the association between platinum resistance and HPV status in cervical cancer using organoid models
Source: BMC Biol. 2026 Jun 26;24:197. doi: 10.1186/s12915-026-02665-w (PMC13591797; doi:10.1186/s12915-026-02665-w)

Figure S2 (related to Figure 3)

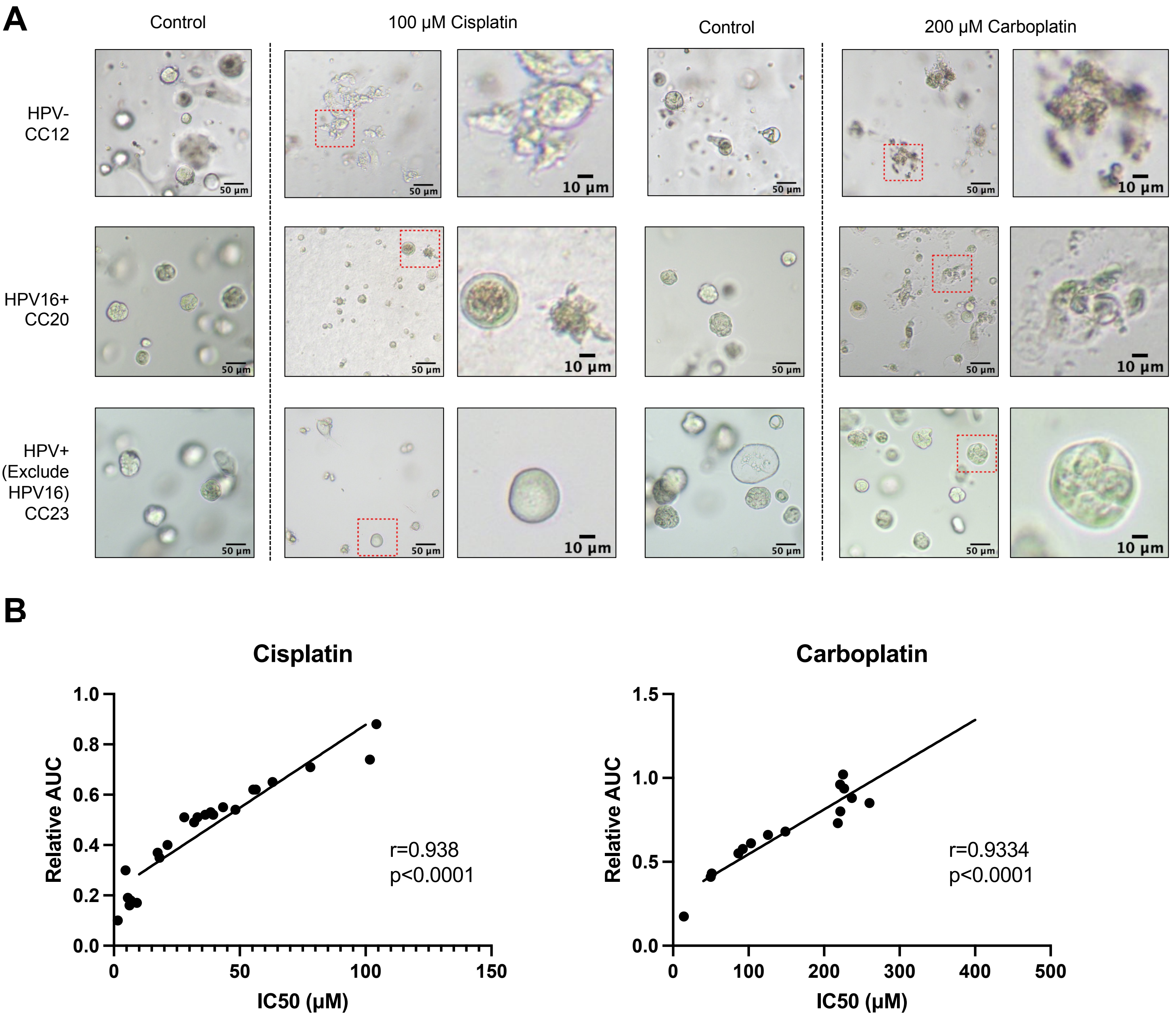

Supplement: Supplementary file 2 [file 12915_2026_2665_MOESM2_ESM.pdf]
